# IGF-1 and IGF-Binding Proteins and Bone Mass, Geometry, and Strength: Relation to Metabolic Control in Adolescent Girls With Type 1 Diabetes

**DOI:** 10.1359/jbmr.080713

**Published:** 2008-07-28

**Authors:** Laurie J Moyer-Mileur, Hillarie Slater, Kristine C Jordan, Mary A Murray

**Affiliations:** 1 Center for Pediatric Nutrition Research, Department of Pediatrics, University of UtahSalt Lake City, Utah, USA; 2 Division of Nutrition, College of Health, University of UtahSalt Lake City, Utah, USA; 3 Division of Endocrinology, Department of Pediatrics, University of UtahSalt Lake City, Utah, USA

**Keywords:** metabolic control, bone, adolescence, diabetes

## Abstract

Children and adolescents with poorly controlled type 1 diabetes mellitus (T1DM) are at risk for decreased bone mass. Growth hormone (GH) and its mediator, IGF-1, promote skeletal growth. Recent observations have suggested that children and adolescents with T1DM are at risk for decreased bone mineral acquisition. We examined the relationships between metabolic control, IGF-1 and its binding proteins (IGFBP-1, -3, -5), and bone mass in T1DM in adolescent girls 12–15 yr of age with T1DM (*n* = 11) and matched controls (*n* = 10). Subjects were admitted overnight and given a standardized diet. Periodic blood samples were obtained, and bone measurements were performed. Serum GH, IGFBP-1 and -5, glycosylated hemoglobin (HbA_1c_), glucose, and urine magnesium levels were higher and IGF-1 values were lower in T1DM compared with controls (*p* < 0.05). Whole body BMC/bone area (BA), femoral neck areal BMD (aBMD) and bone mineral apparent density (BMAD), and tibia cortical BMC were lower in T1DM (*p* < 0.05). Poor diabetes control predicted lower IGF-1 (*r*^2^ = 0.21) and greater IGFBP-1 (*r*^2^ = 0.39), IGFBP-5 (*r*^2^ = 0.38), and bone-specific alkaline phosphatase (BALP; *r*^2^ = 0.41, *p* < 0.05). Higher urine magnesium excretion predicted an overall shorter, lighter skeleton, and lower tibia cortical bone size, mineral, and density (*r*^2^ = 0.44–0.75, *p* < 0.05). In the T1DM cohort, earlier age at diagnosis was predictive of lower IGF-1, higher urine magnesium excretion, and lighter, thinner cortical bone (*r*^2^ ≥ 0.45, *p* < 0.01). We conclude that poor metabolic control alters the GH/IGF-1 axis, whereas greater urine magnesium excretion may reflect subtle changes in renal function and/or glucosuria leading to altered bone size and density in adolescent girls with T1DM.

## INTRODUCTION

Good bone health is important to the maintenance of functionality in the aging population. Pubertal bone mineral accretion is predictive of osteoporosis in aging women.(1) Recent observations suggest that children and adolescents with type 1 diabetes mellitus (T1DM) are at risk for decreased bone mineral acquisition.(2–7) Our research group has observed significantly lower bone mass in adolescents with T1DM compared with a nondiabetic reference population.(8,9) Children and adolescents with poorly controlled T1DM are at risk for decreased bone mass. Disease duration,(7,10) poor metabolic control,(2,6,11,12) and diabetic complications such as retinopathy or neuropathy(2,10) are reported to have a negative impact on bone mass, although others have found no relationship.(13,14) Poor blood glucose control has been associated with lower bone mass in adults and adolescents with T1DM(8–11) but not in children.(3,14)

The growth hormone (GH)/IGF axis is a major determinant of bone mass acquisition. Circulating IGF-1 is produced by the liver, is structurally similar to insulin,(15) and helps to mediate the skeletal growth promoting actions of GH. IGF-1 is also produced locally by muscle and bone tissue, where it is hypothesized to act in a paracrine manner. Along with systemic GH and estradiol, local bone IGF-1 concentrations are regulated by PTH, 1,25-dihydroxyvitamin D_3_, and other cytokines and growth factors.(16) IGF-1 functions as a key anabolic regulator of bone cell activity by decreasing collagen degradation and increasing bone matrix deposition and osteoblastic cell recruitment.(15–17) In healthy children, serum IGF-1 concentrations correlate well with BMC.(18) In adolescents with poorly controlled T1DM, there is established evidence of increased secretion of GH but low levels of IGF-1 compared with matched controls.(13,17) Thus, the GH/IGF axis has received considerable attention as a mechanism for inadequate bone formation in T1DM.(19–22)

IGF binding proteins (IGFBPs) control the tissue availability of IGF-1 and therefore are major regulators of IGF-1 action. IGFBP-3 is the predominant circulating IGFBP, binding >95% of circulating IGF. Production of IGFBP-3 is stimulated by GH, whereas IGFBP-1 is increased in the absence of GH. In patients with osteoporosis, IGFBP-1 and IGFBP-4 inhibit IGF bone cell proliferation by sequestering IGF-1 and preventing binding to the IGF receptor.(15) IGFBP-3 and IGFBP-5 facilitate IGF action in bone cells; bone matrix proteoglycans do not bind IGFs in the absence of IGFBP-3 and IGFs do not bind to hydroxyapatite in the absence of IGFBP-5.(15) There is also some evidence that IGFBPs regulate bone formation independent of IGF function. For example, IGFBP-5 has been found to stimulate markers of bone formation in osteoblasts that lack functional IGF.(17)

There is a paucity of information on how IGF-1 and IGFBPs regulate skeletal growth in children with T1DM during puberty. The purpose of this study was to assess the GH/IGF axis and bone health in adolescent girls with T1DM. A secondary objective was to examine the relationships between IGF-1 and its binding proteins and the characteristics and markers of bone turnover. We tested the central hypothesis that T1DM is associated with a state of partial GH resistance resulting in decreased IGF action and altered IGFBP activity, leading to diminished bone size, density, and strength.

## MATERIALS AND METHODS

### Subjects

Girls 12–15 yr of age with T1DM for a minimum of 12 mo (*n* = 11) were recruited from the Utah Diabetes Center Pediatric Program, Salt Lake City, UT, USA. Healthy girls matched for race, age, and maturation were recruited as controls (*n* = 10). Exclusion criteria included poor metabolic control (glycosylated hemoglobin [HbA_1c_] > 9.0%), hypertension (diastolic blood pressure > 90th percentile for age), microalbuminuria, hypo- or hyperthyroidism, GH deficiency, celiac disease (tTG ≥7.0 AU or symptoms) or other health conditions or medication use known to alter growth or bone mineral deposition. This study was approved by the University of Utah Institutional Review Board for Human Subjects.

### Protocol

**FIG. 1:**
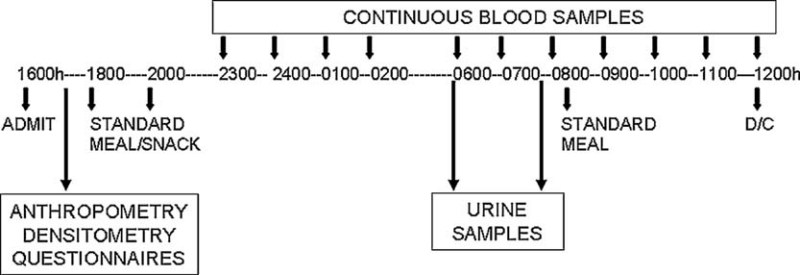
Protocol schema. Subjects were admitted to the General Clinical Research Center at 5:00 p.m. and received standardized meals at 6:00 p.m., 8:00 p.m., and 8:00 a.m. Hourly blood samples were obtained from 11:00 p.m. to 11:00 a.m., and urine samples were obtained at 6:00 and 8:00 a.m.

The schema for the protocol is shown in Fig. 1. Potential subjects were evaluated in a prestudy visit. Appropriate subjects were admitted to the General Clinical Research Center (GCRC) at 4:00 p.m. on the day of study. Written informed consent and assent were obtained at admission. An intravenous catheter was placed in a forearm vein for blood access by 10:00 p.m., and blood samples were obtained from 11:00 p.m. to 11:00 a.m. First and second morning voids of urine were collected at 6:00 and 8:00 a.m. Subjects were given standardized meals and snacks with energy based on the subject's sex, age, and body weight and a substrate distribution of 15% protein, 35% fat, and 50% carbohydrate. T1DM subjects received their usual insulin dose.

Each study participant completed a health history questionnaire, which included a family and personal medical history and current medication use. Pubertal maturation was determined by a pediatric endocrinologist using Tanner stage criteria.(23) Calcium intake(24) and past-year physical activity(25) were also assessed by questionnaire. Metabolic hours of weight-bearing physical activity for the previous year were calculated by assigning each weight-bearing activity a number representing metabolic cost (MET).(26) Height without shoes was measured to the nearest 0.1 cm for each participant using a Height-Rite Stadiometer (Model 225; Seca, Culver City, CA, USA), and weight was measured to the nearest 0.1 kg by digital scale (Model 5002; Scan-Tronix, Carol Stream, IL, USA). Body mass index (BMI, kg/m^2^) was calculated for all subjects.

### Bone measurements

Three cross-sectional slices of the nondominant tibia were measured by pQCT (XCT-2000; Stratec/Orthometrix, White Plains, NJ, USA) at relative distances of 4%, 38%, and 66% from the distal tibia growth plate to assess trabecular and cortical bone and the muscle cross-sectional area (CSA), respectively. Dominance and nondominance were determined by asking whether the subject was right or left handed. Tibia metaphyseal bone properties, including trabecular volumetric BMD (vBMD; mg/cm^3^), were assessed from the 4% CSA. The 38% CSA was used to assess tibia diaphyseal bone properties, including cortical bone vBMD, BMC (mg), and geometric bone properties: bone CSA (mm^2^), cortical CSA (bone CSA less marrow CSA), marrow CSA (bone CSA less cortical CSA), and cortical thickness (mm). Muscle CSA and the polar strength strain index (pSSI, mm^3^) were determined from the 66% distal cross-section to examine bone muscle relationship(27) and bone strength.(28) Analysis parameters and modes were as previously described.(29) In addition, whole body bone area (BA, cm^2^), BMC (g), lean body mass (LBM, kg), percent body fat, femoral neck (FN) and lumbar spine (LS) BA, BMC, and areal BMD (aBMD, g/cm^2^) were determined by DXA (4500A; Hologic, Waltham, MA, USA). Height for age, whole body BA to height, and whole body BMC to BA were assessed to determine whether bone mass was reduced because of short, narrow, or lighter bones.(30) FN and LS BMC and BA values were used to determine bone mineral apparent density (BMAD, g/cm^3^).(31) The CV for repositioning in adult volunteers was <2.5% for trabecular and cortical bone vBMD using pQCT and <1.0% for aBMD measured by DXA in our laboratory. The daily CVs for calibration phantoms were 0.1% and 0.3% for pQCT and DXA, respectively. The same experienced radiology technician performed all measurements. Although radiation exposure was minimal (≍≈21.5 μSV total), pregnancy tests were performed before densitometry on all girls with Tanner stage ≥2.

### Biochemical measures

GH, IGF-1, insulin, and glucose levels were determined from samples obtained hourly from 11:00 p.m. to 6:00 a.m. and IGF-1 and IGFBP-1 from 6:00 a.m. to 10:00 a.m. The area under the curve (AUC) was calculated for 11:00 p.m. to 6:00 a.m. values to assess the GH/IGF axis activity and between 9:00 and 10:00 a.m. to examine postprandial differences between IGF-1 and IGFBP-1. IGFBP-3, -4, and -5 values. All other serum values were determined from the fasting 8:00 a.m. blood sample. Pyridinoline (Pyd), deoxypyridinoline (Dpd), and hydroxylysine-derived cross-links of mature collagen degradation were selected to indirectly assess bone resorption. Pyd and Dpd cross-links were measured from the 6:00 a.m. urine sample, whereas calcium, phosphorus, magnesium, and creatinine assays were determined on the 8:00 a.m. sample. Serum samples (10 ml) were collected using a standard technique from indwelling catheters without anticoagulants and were processed to avoid hemolysis. Serum was separated by centrifugation and stored at –70°C until analysis. Urine samples were collected without preservative and stored at –70°C. ELISA assays were used to measure GH, IGF-1, IGFBP-1, -3, and -4, insulin, 1,25(OH)_2_ vitamin D (DSL), and bone-specific alkaline phosphatase (BALP; Metra); high performance liquid chromatography (HPLC) was used to measure HbA_1c_; and the spectrophotometric technique was used to measure serum and urine minerals. IGFBP-5 levels were measured by the Musculoskeletal Disease Center Laboratory, Loma Linda University, using polyclonal guinea pig antiserum and recombinant IGFBP-5 as standard and tracer, respectively, Pyd and Dpd were measured by HPLC, with standards purchased from Quidel (San Diego, CA, USA). Urinary Pyd cross-link concentration was normalized to urinary creatinine (Beckman Creatinine Analyzer 2) and calculated as μmol/mol creatinine. Measurements were made in duplicate, and the average values were entered into the database.

### Statistical methods

Statistical analyses included independent *t*-test and χ^2^ to identify differences in demographic variables of interest between T1DM and control subjects. Standard deviation scores (SDSs) were generated for body weight and height using EpiInfo.(32) The ratios of IGFBP-1, -3, -4, and -5 to IGF-1 were calculated. Multivariate and repeated-measures analysis of covariance (MANCOVA) and posthoc tests were used to compare means between T1DM and control groups for blood and urine results, bone measurements, and body composition using pubertal stage and height SDS as covariates. Bone results are reported as adjusted means with the 5th and 95th CIs. Correlation analysis was run between selected continuous variables. Stepwise linear regression was performed to identify which variable predicted biochemical and bone characteristics. Statistical analyses for these data were performed using the SPSS-PC+ (Version 13.1; SPSS) statistical software program with significance set at *p* ≤ 0.05.

## RESULTS

Demographic data are presented in Table 1. Age, pubertal maturation, body size and composition, reported calcium intake, and physical activity levels were similar between groups.

**Table 1:** Subject Demographics

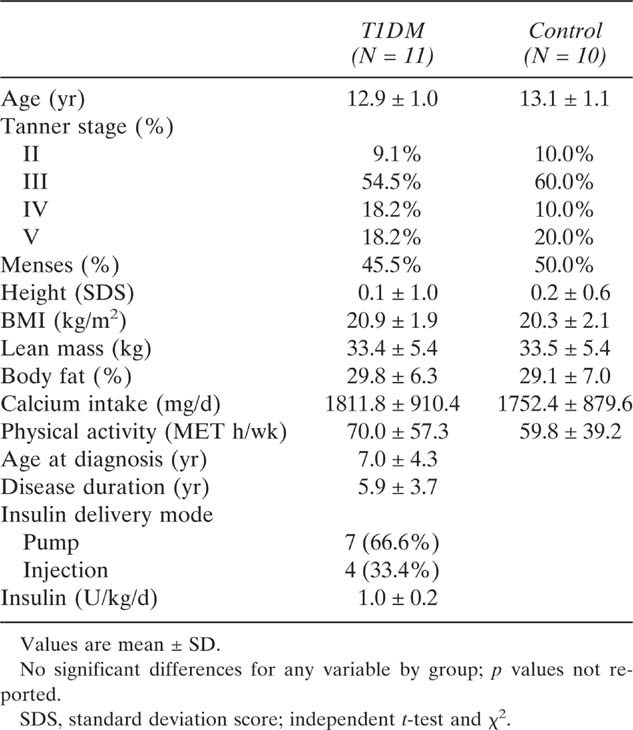

### Biochemical findings

**FIG. 2:**
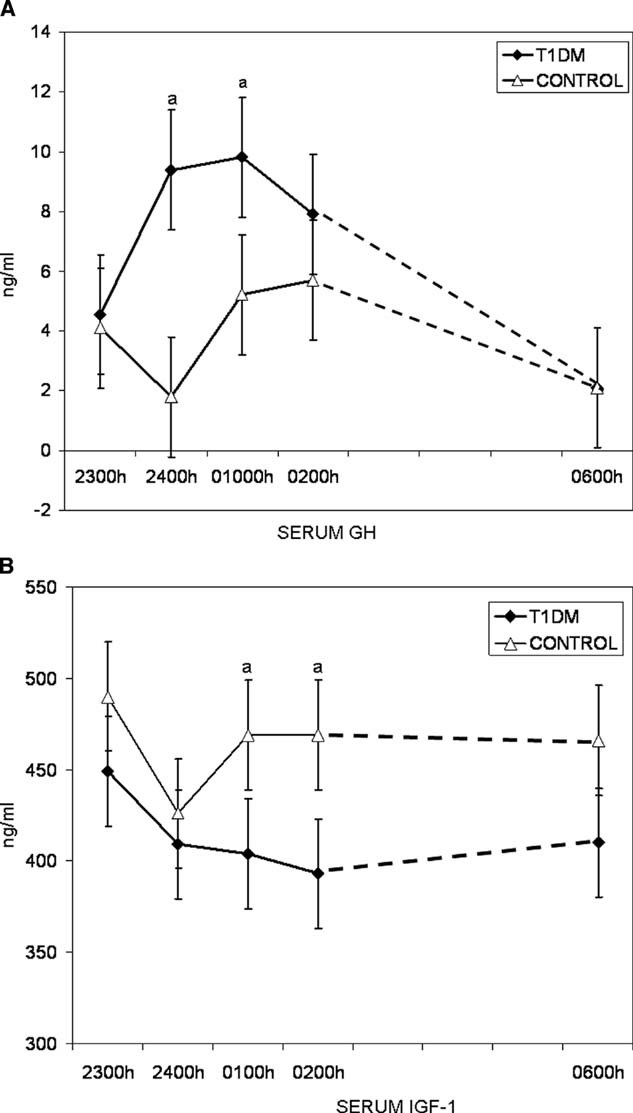
(A and B) Mean serum GH and IGF-1 levels between 11:00 p.m. and 6:00 a.m. for T1DM (▪) and control (▵) groups. Absence of IGF-1 rise in response to elevated GH confirms an altered GH/IGF-1 axis response in T1DM. ^a^*p* ≤ 0.05.

**FIG. 3:**
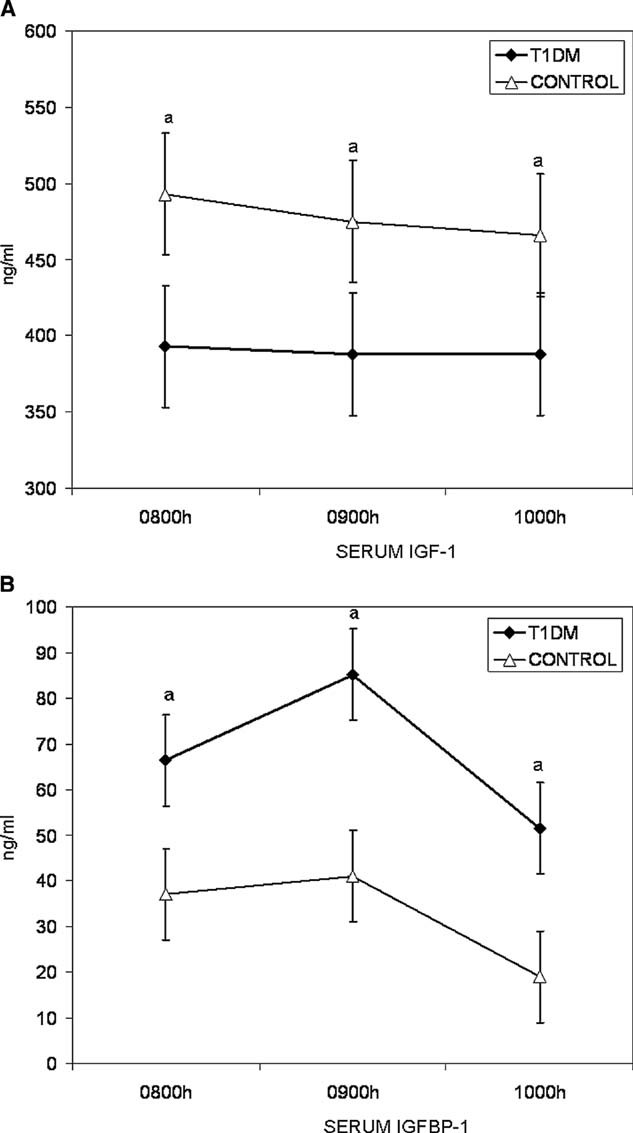
(A and B) Mean postprandial serum IGF-1 and IGFBP-1 levels for T1DM (▪) and control (▵) groups. Lower IGF-1 and higher IGFBP-1 values confirm sustained alteration in GH/IGF-1 axis in T1DM. ^a^*p* ≤ 0.05.

GH/IGF-1 axis results were as expected (Table 2). The rise in GH levels was greater at 12:00 p.m. and 1:00 a.m. and the IGF-1 response was lower in T1DM girls compared with controls (*p* < 0.05; Fig. 2). The mean AUC values for GH and IGF-1 from 11:00 p.m. to 6:00 a.m., however, were not statistically significant. The mean hourly values for serum insulin and glucose at 12:00 p.m. to 6:00 a.m. were significantly higher in T1DM girls (*p* < 0.05) than controls. The mean hourly postprandial values for IGF-1 and IGFBP-1 are plotted in Fig. 3. Differences are again evident with lower mean IGF-1 and higher IGFBP-1 values found in the T1DM cohort compared with controls at 8:00, 9:00, and 10:00 a.m. (*p* < 0.05). This finding is substantiated by lower IGF-1 and higher IGFBP-1 AUC values in T1DM girls from 8:00 to 10:00 a.m. (778.6 ± 232.3 ng/dl IGF-1 and 169.7 ± 84.4 ng/dl IGFBP-1 T1DM versus 954.8 ± 198.9 ng/dl IGF-1 and 79.5 ± 46.3 ng/dl IGFBP-1 controls; *p* ≤ 0.05). The mean fasting levels for IGBP-3, -4, and -5 were similar (Table 2). The ratios for IGFBP-1, -3, -4, and -5 relative to IGF-1, however, were higher in T1DM girls, although only IGFBP-1 and -5/IGF-1 ratios were statistically significant (*p* ≤ 0.05; data not shown).

**Table 2:** Serum and Urine Results^*^

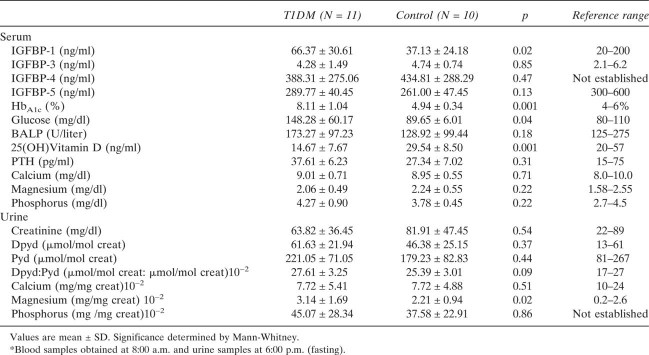

Additional serum and urine results are also presented in Table 2. T1DM girls had higher fasting HbA_1c_ and serum glucose values versus controls (*p* ≤ 0.01). The mean values for markers of bone turnover tended to be greater, as evidenced by higher serum BALP and urine Dpd/Pyd levels and lower mean 25(OH) vitamin D levels in T1DM girls. Urine magnesium levels were markedly higher in T1DM girls compared with controls (*p* < 0.05). We did not detect differences in serum PTH, calcium, magnesium, and phosphorus levels or urine calcium and phosphorus excretion. With the exception of serum HbA_1c_, glucose, and urine magnesium for T1DM, all values were within the normal reference range.

### Skeletal characteristics

The whole body, FN, LS, and tibial bone geometry, density, and strength findings for T1DM and control groups are presented in Table 3. Whole body BMC/BA and FN aBMD and BMAD values were significantly lower in T1DM girls (*p* ≤ 0.05). The evaluation of tibial bone geometry and density showed significantly lower cortical BMC and cortical BMC/muscle CSA (*p* < 0.05), with a trend toward thinner cortical bone thickness and vBMD (*p* = 0.07 and 0.09, respectively) in T1DM girls. All other skeletal findings were similar between groups.

**Table 3:** Skeletal Characteristics

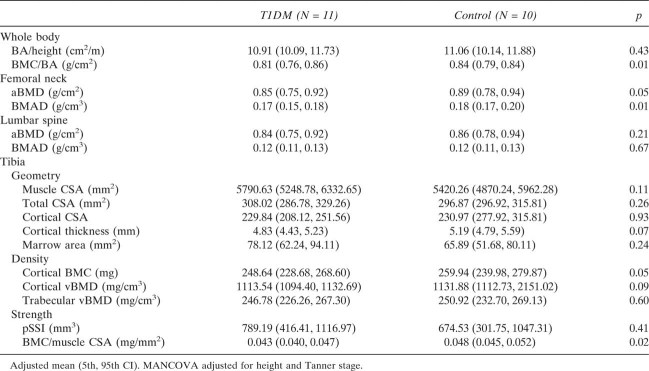

### Predictors

Poor metabolic control predicted lower IGF-1 AUC values, contributing 50–94% of the total variability in fasting and postprandial levels, respectively (*p* ≤ 0.01). Poor metabolic control was also correlated with higher IGFBP-1 and -5/IGF-1 ratios (*R* = 0.65, *p* ≤ 0.01), but was not a significant predictor for either IGFBP-1 or -5 in the regression model. Greater urine magnesium excretion was predictive of a higher IGFBP-3/IGF-1 ratio, accounting for 26% of the variability (*p* = 0.001). The onset of menses was predictive of a higher IGFBP-4/IGF-1 ratio (*r*^2^ = 0.23, *p* = 0.001). The absence of menses, higher serum 25(OH) vitamin D levels, and poor metabolic control predicted higher levels of BALP (*r*^2^ = 0.69, *p* = 0.001), accounting for 32%, 28%, and 9% of the variance, respectively. Higher 25(OH) vitamin D levels were predicted by higher BALP and lower GH levels, which accounted for 28% and 13% of variability, respectively (*p* < 0.01). Higher serum glucose was the single predictor of greater bone resorption (*r*^2^ = 0.48, *p* = 0.001) and urine magnesium excretion (*r*^2^ = 0.61, *p* < 0.01), accounting for 48% and 61%, respectively, of the variability observed in Dpd/Pyd and urine magnesium levels.

The relationships between T1DM disease–related factors and biochemical findings were examined by removing control and adding age at diagnosis, disease duration, and insulin dose (U/kg/d) to the regression model. A younger age at diagnosis was predictive of lower IGF-1 and higher IGFBP-1/IGF-1 ratios and greater urine magnesium excretion, accounting for 79–97% of the variability. Poor metabolic control strongly correlated with the ratios of IGFBP-4 and -5 to IGF-1 (*R* = 0.78 and 0.65, respectively, *p* < 0.01), but was only predictive of IGFBP-5/IGF in the regression model (*r*^2^ = 0.91, *p* = 0.001), accounting for 72% of the variance.

The relationships between T1DM disease–related factors and skeletal findings were examined by removing control and adding age at diagnosis, disease duration, and insulin dose (U/kg/d) to the regression model. Longer disease duration was predictive of smaller whole body bone size and lower tibia total and cortical CSA, cortical BMC, and thickness (*r*^2^ ≥ 0.65, *p* = 0.001), accounting for 6.9–80% of the variance.

Higher urine magnesium excretion was predictive of a greater height deficit, lighter bones, decreased FN BMAD, lower tibia cortical bone CSA, thickness, BMC, and tibia marrow CSA (*r*^2^ = 0.26–0.84, *p* ≤ 0.05) and accounted for 26–60% of the variance. A larger ratio of IGFBP-1 relative to IGF-1 was predictive of smaller bones and accounted for 36% of the variability observed in the BA/height ratio (*p* = 0.001). Trabecular vBMD was inversely related to IGFBP-1 and -5/IGF-1 ratios (*r*^2^ = 0.40, *p* = 0.001), accounting for 11% and 29% of the variance, respectively. Greater tibia bone strength (pSSI) was predicted by higher IGF-1 levels (*r*^2^ = 0.23, *p* = 0.001).

## DISCUSSION

To our knowledge, this is the first study directed at the relationship between metabolic control, GH/IGF-1 axis activity, and bone health in adolescent girls with T1DM. The altered nighttime GH/IGF axis activity observed in our study confirms the work of others. We also found significantly higher IGFBP-1 and -5 levels and less overall bone mineral deposition in adolescent girls with T1DM compared with healthy matched controls. Metabolic control was the predominant predictor of GH/IGF-1 axis alterations, whereas higher urine magnesium excretion was the predominant predictor of whole body and cortical bone deficits.

During puberty, sex hormones induce an increase in the GH/IGF system to promote linear growth and bone expansion. As maturation progresses, bone turnover is reduced, which increases cortical bone thickness and strength.(27,33) We showed an alteration in the GH/IGF system and a negative association between IGFBP-1 and bone size in adolescent girls with T1DM. Although only nighttime GH/IGF-1 axis activity was assessed in our study, daytime GH/IGF-1 axis activity mimics nighttime GH/IGF-1 axis activity and increases insulin resistance and exogenous insulin requirements in adolescent girls with T1DM.(34) The significantly lower postmeal IGF-1 and higher IGFBP-1/IGF1 levels in the T1DM cohort also suggest that GH/IGF-1 axis activity remains altered throughout the day.

Our group and others have previously reported skeletal deficits in adolescents with T1DM.(2–12) In our previous work, lower whole body BMC, FN densities, trabecular vBMD values,(8) and BMC acquisition(9) were predicted by poor metabolic control.(8,9) In animal and in vitro cell models, chronic hyperglycemia is linked to altered osteoblast differentiation and maturation.(15,16) Recently, Thrailkill(35) showed preservation of bone formation by insulin administration during osteogenesis in a mouse model. Neither insulin treatment nor glucose concentration, however, entirely explain lower bone mass in adolescents with T1DM.

In this study, adolescent girls with T1DM were matched for age, body size, and pubertal maturation with healthy controls. Both cohorts reported similar levels of dietary calcium intake and weight-bearing activities. We again observed significantly lower whole body BMC relative to BA and FN bone density and decreased cortical bone BMC relative to muscle in adolescent girls with T1DM. Furthermore, we noted a trend toward thinner tibia cortical thickness and decreased cortical vBMD in the T1DM cohort. Reduced radius cortical vBMD and total, cortical, and muscle CSA has been reported in children and adolescents diagnosed with T1DM before the age of 5.(7) A strong negative association between an earlier manifestation of T1DM and smaller bone size and diminished BMC was also shown in our T1DM cohort.

Both FN aBMD and BMAD values were lower in our T1DM cohort compared with controls. Significantly lower size-adjusted FN and LS aBMD has recently been reported in adolescent and young adult women with T1DM(36) and may explain, in part, the higher hip fracture incidence observed in adults with childhood onset T1DM.(37) We did not detect, however, LS aBMD or BMAD deficits in our T1DM cohorts in our present or previous studies.(8,9) Similar lumbar spine results have been found in children with recent onset of T1DM (<3 yr) and good metabolic control (HbA_1c_ < 8.2%).(14) The vertebrae, or lumbar spine, are primarily composed of trabecular bone, and decreased trabecular vBMD is associated with poor metabolic control.(8,9,11) The absence of trabecular bone deficits in our T1DM cohort may be explained by restriction of enrollment to girls with HbA_1c_ values of ≤9% for the year before study.

Overall metabolic control was not associated with skeletal findings but did predict alterations in the GH/IGF-1 axis. Lower IGF-1 and greater IGFBP-1 and -5 relative to IGF-1 levels were predicted by poor metabolic control, whereas a higher IGFBP-1/IGF-1 ratio was predictive of smaller, less mineralized bones. Increased urine magnesium loss is associated with decreased stature and linear growth in T1DM.(38) Magnesium depletion in animals is characterized by impaired bone growth, decreased osteoblast number, increased osteoclast number, and loss of trabecular bone.(38) Microalbuminuria is associated with greater urine magnesium loss and lower serum 25(OH) vitamin D levels in adolescents and young adults with T1DM.(39) Our T1DM cohort was prescreened to rule out the presence of microalbuminuria to help exclude subjects with diabetic nephropathy. Diabetic microangiopathy, however, seems to precede the elevation of albumin excretion by 3 yr in adolescents with T1DM.(40) Despite serum glucose levels below the renal threshold to elicit glucosuria (<200 mg/dl), greater urine magnesium loss was associated with elevated serum glucose levels in this study. Therefore, greater magnesium excretion may reflect subtle changes in renal tubular function and/or glucosuria, which subsequently impaired skeletal growth in our T1DM subjects.

The cross-sectional design and pubertal maturation range of our study population limit the interpretation of our results. Although we attempted to compensate by using pubertal stage–matched controls, the majority of subjects were in the later phase of puberty, when most pubertal-driven skeletal growth has occurred but before completion of skeletal mineralization. Whereas the GH-IGF axis abnormalities in diabetes are well characterized, there is little understanding of how these abnormalities effect skeletal growth at the tissue level. IGFBPs profiles are tissue specific, and serum IGFBPs measurements may not reflect their modulation of IGF-1 in bone. Furthermore, we did not separate the effects of estrogen on the GH-IGF axis, both of which contribute to skeletal growth and mineralization during puberty.

In summary, poor metabolic control predicted alterations in the GH/IGF-1 axis and IGFBP levels, whereas greater urine magnesium excretion predicted variations in bone geometry and density in adolescent girls with T1DM. Additional studies are needed to gain insights about bone formation and its dysregulation in pubertal children with T1DM.
